# Vigilant conservatism in evaluating communicated information

**DOI:** 10.1371/journal.pone.0188825

**Published:** 2018-01-10

**Authors:** Emmanuel Trouche, Petter Johansson, Lars Hall, Hugo Mercier

**Affiliations:** 1 Department of Psychology, Yale University, New Haven, Connecticut, United States of America; 2 Lund University Cognitive Science, Lund University, Lund, Sweden; 3 Swedish Collegium for Advanced Study, Uppsala University, Uppsala, Sweden; 4 Institut des Sciences Cognitives—Marc Jeannerod, CNRS, Bron, France; Eberhard-Karls-Universitat Tubingen Medizinische Fakultat, GERMANY

## Abstract

In the absence of other information, people put more weight on their own opinion than on the opinion of others: they are conservative. Several proximal mechanisms have been suggested to account for this finding. One of these mechanisms is that people cannot access reasons for other people’s opinions, but they can access the reasons for their own opinions—whether they are the actual reasons that led them to hold the opinions (rational access to reasons), or post-hoc constructions (biased access to reasons). In four experiments, participants were asked to provide an opinion, and then faced with another participant’s opinion and asked if they wanted to revise their initial opinion. Some questions were manipulated so that the advice participants were receiving was in fact their own opinion, while what they thought was their own opinion was in fact not. In all experiments, the participants were consistently biased towards what they thought was their own opinion, showing that conservativeness cannot be explained by rational access to reasons, which should have favored the advice. One experiment revealed that conservativeness was not decreased under time pressure, suggesting that biased access to reasons is an unlikely explanation for conservativeness. The experiments also suggest that repetition plays a role in advice taking, with repeated opinions being granted more weight than non-fluent opinions. Our results are not consistent with any of the established proximal explanations for conservatism. Instead, we suggest an ultimate explanation—vigilant conservatism—that sees conservatism as adaptive since receivers should be wary of senders’ interests, as they rarely perfectly converge with theirs.

## Introduction

Are individuals able to properly take into account novel information? Given how crucial this task is to form an accurate representation of one’s environment, humans, along with other cognitively complex animals, should be able to perform it well. Some computational costs are necessarily involved, hence behavior needs not be perfectly in line with normative models, such as Bayes’ rule, but it should not be consistently biased. For instance, there should not be a general bias to favor one’s prior beliefs over novel information.

Some results suggest that, when asked to revise probability estimates in light of novel information, participants tend to put too much weight on the base rates—their prior beliefs—and not enough on novel information (e.g. [1]). This tendency has been described as conservatism [2]. However, in other cases participants significantly underweight base rates [3]. There is thus, in this area, no consistent bias towards conservatism.

Yet one area of research has demonstrated a consistent bias towards conservatism: the field of advice taking ([4,5] for reviews of the advice taking literature, see [6,7]). This field studies how individuals aggregate opinions, often their own and that of others. Strictly speaking, the vast majority of the studies do not deal with advice per se (i.e. an opinion that is provided with the intent of helping someone make a decision or a judgment), since the opinions to be aggregated have not been generated as pieces of advice, and they are not presented as pieces of advice, merely as the opinions of other individuals (for some exceptions, see [8–12]). Still, for reasons of clarity and consistency, we will adopt here the common vocabulary of *judge* to refer to the individual who aggregates opinions, of *advisor* to refer to the individual who provides the opinions aggregated by the judges, and of *advice* to refer to these opinions.

A robust finding from the study of advice taking is that judges heavily discount advice, putting more weight on their own opinion. Typically, judges adjust their opinion between 20% and 30% towards the advisor’s opinion, so that if they started with 0, and the advice was 100, the average answer after aggregation would fall between 20 and 30 (e.g. [5]). This is often referred to as ‘egocentric discounting’ [5], but here we will adopt the more general term of conservatism. An elegant demonstration of this conservatism was provided by Yaniv and Choshen-Hillel [13], who showed that advisors’ opinions are taken into account in a largely unbiased manner when the advisors haven’t been able to form their own opinion prior to seeing the advisors’ opinions.

Conservatism has often been explained in terms of differential access to reasons—judges can access their own reasons, but not those of the advisors—although other factors seem to also be at play (see, e.g. [13,14]). The main goal of this article is to provide confirming evidence that neither rational nor biased access to reasons can entirely account for conservatism. Two secondary goals are, first, to provide tentative evidence in favor of an ultimate explanation for conservatism—which we dub vigilant conservatism—and, second, to introduce the methodology of choice blindness as a tool to study advice taking.

### Proximal explanations for conservatism

Several explanations have been offered for conservatism in advice taking. All of these explanations are concerned with the proximal level: describing the psychological mechanisms that purportedly cause conservatism. One of the most prominent of these proximal explanations is that judges can more easily access the reasons supporting their opinions than those supporting the advisor’s opinion [5]. This explanation can take two forms: a rational one (*rational access to reasons*) and a biased one (*biased access to reasons*). Rational access to reasons rests on the genuine informational asymmetry, for the judge, between her own reasons and that of the advisor. Because of this genuine asymmetry, it is largely rational for judges to discount advisors’ unsupported opinions. Rational access to reasons predicts that, when participants have less access to the reasons that motivated their opinion, then they should be less conservative. Soll and Mannes [14] have shown that when judges aggregate opinions, the absence of the cues on which they initially grounded their opinion does not diminish conservatism, thus arguing against rational access to reason.

Biased access to reasons takes place when judges search for reasons that support their prior opinions, irrespective of whether these were the reasons that originally led them to form the opinions. By contrast, rational access to reasons entails that judges rely on the actual reasons that led them to defend an opinion in the first place. Many experiments have shown that individuals have a tendency to rationalize their opinions (e.g. [15,16]), and it is thus very likely that biased access to reasons is at play in advice taking.

Another explanation for conservatism is that when judges have to take advice into account, their prior opinion could play the role of an anchor that biases how they access information in a way that is consistent with their prior opinion (e.g. [17]). A potential issue with this explanation is that anchoring typically only has short-term effects (for review, see [4]). However, judges are found to be conservative even if they have had to perform several tasks between the time they initially provided their opinion and the time they have to take the advice into account (e.g. [4]).

The final explanation we consider here is that of *egocentric beliefs* (see [14]). Due to the operation of motivated reasoning [18], individuals often form inflated views of their own abilities (e.g. [19]). If judges believe, even in the absence of good evidence, that their abilities are superior to those of advisors, this could explain why judges would tend to favor their own opinions. Soll and Mannes [14] have tracked both how judges aggregated opinions, and how they rated their own abilities and that of the advisors. Although they found that judges consistently overrated their ability relative to that of the advisors, once this bias was controlled for, the judges were still conservative. It thus seems that conservatism cannot be entirely accounted for by judge’s inflated views of their own abilities.

### An ultimate explanation for conservatism

Although conservatism is maladaptive as a general strategy for updating beliefs, it can be adaptive in specific cases. In particular, communicated information should be distinguished from information gathered through perception. Our own perceptual mechanisms have evolved to provide us with reliable information. By contrast, other individuals have their own incentives, which rarely match ours perfectly, and they might provide us with unreliable information.

It has been suggested that humans are endowed with a set of cognitive mechanisms designed to minimize the risks of taking unreliable communicated information into account—mechanisms of epistemic vigilance [20]. One of these mechanisms consists in checking whether some communicated information is coherent with one’s prior beliefs [21,22]. If there is an inconsistency, one should then be conservative and favor one’s prior beliefs, since the communicated information is, everything else equal, more likely to be unreliable. This provides an evolutionary explanation for the conservatism observed in the advice taking literature, which exclusively deals with communicated information.

This conservatism, however, can preclude judges from making the best of communicated information. Within the epistemic vigilance framework, two mechanisms can overcome conservatism: trust and argumentation. If judges think that the novel communicated information comes from an advisor who is honest and particularly competent, they should be able to overcome their conservatism. Indeed, advisor expertise, as well as other cues to trustworthiness, have been shown to reduce conservatism (e.g. [8,11,23–25]). Advisors can also offer arguments in defense of their views. If the judge deems the arguments sufficiently strong, she should also be able to overcome her conservatism. Several experiments have shown that allowing judges and advisors to exchange arguments has this effect (e.g. [26–29]).

The epistemic vigilance framework thus provides the following explanation for conservatism. When one is confronted with communicated information that clashes with one’s prior beliefs, and in the absence of other cues to advice reliability—such as high advisor expertise or strong arguments—it is adaptive to favor one’s prior beliefs—i.e. to be conservative. We dub this explanation *vigilant conservatism*. Vigilant conservatism is thus the hypothesis that we have an evolved bias to discount communicated information by default, in the absence of cues to the contrary.

All of the explanations reviewed previously were concerned with the proximal level of explanation. As such, they are not intrinsically incompatible with vigilant conservatism, which is an ultimate explanation, an explanation of what the function of conservatism is. This function could be served by any number of psychological mechanisms. Still, proximal and ultimate explanations can also make incompatible predictions. In particular, vigilant conservatism predicts that we should observe conservatism even in some cases that cannot be accounted for by any of the proximal explanations offered so far. For instance, the conditions for vigilant conservatism to apply still exist when judges have not accessed reasons for their opinion, and when they do not think they are more competent than the advisors.

The present experiments set up situations in which proximal explanations for vigilant conservatism—in particular rational access to reason, but also biased access to reason, and to a lesser extent, egocentric beliefs—cannot account for conservatism. If vigilant conservatism is correct, participants should still be conservative.

### The choice blindness paradigm

We relied on the choice blindness paradigm (see, e.g. [16,30]). In this paradigm, participants first make a decision, or answer a question or series of questions. They are then asked to perform a new task that involves their previous answers, such as justifying them. However, one or several of their answers are manipulated so that participants are told that they have answered something different from their actual answer. Across several experiments, most participants have been shown to often not notice such manipulations. Participants who did not notice the manipulations could then, for instance, defend an opinion opposite from the one they had stated as their own a few minutes earlier (e.g. [16,30]).

The use of a choice blindness paradigm raises questions regarding which cues people use to ascertain the ownership of a given opinion—whether it is their own opinion or not. When individuals draw their opinions from memory or inference, they can be said to have internal cues that the opinion is their own. But individuals can also rely on external cues to decide that an opinion is their own. For instance, academics—in our experience at least—sometimes consult their previous writings to remember what they thought of topics they do not focus on anymore. When they do so, they rely on external cues—such as their name on the article—to decide which article to consult and thus which opinion to adopt.

To our knowledge the role of external and internal cues in advice taking had only been studied once before, by Harvey and Harries [4]. Judges had to combine the forecasts of four advisors. For one of the four pieces of advice, internal and external cues to ownership were manipulated in a 2*2 design. The internal cue was the advice itself: it was either the opinion of another participant, or the judge’s own previously stated opinion. The external cue consisted in the experimenters telling the judges that the source of the advice was either someone else or the judge himself. As a result, participants could be confronted either with a piece of advice that was someone else’s and labeled as such, someone else’s and labeled as their own, their own and labeled as such, or their own and labeled as someone else’s.

Harvey and Harries [4] found that a single cue to ownership was enough to trigger conservatism. The relevant piece of advice was equally taken into account in all conditions except the one in which both cues indicated that the advice was someone else’s (i.e. when it actually came from someone else and was indicated as such). In this condition, the advice was less taken into account.

In the Harvey and Harries [4] experiment, external and internal cues to ownership were not directly pitted against each other. In particular, participants were never put in a situation in which they were faced with a choice between an opinion which they thought was their own (but which wasn’t), and an opinion which they thought wasn’t their own (but which was). This is what the four present experiments propose to do. The four experiments presented here have a similar design. In the first phase, participants (the judges) are asked to estimate the life expectancy in eight countries. In the second phase, they are told that they will be given the answers of another participant (the advisor) and that they can then provide a new answer. For each question, judges were also reminded of their initial answer. In each experiment, one or two of the questions are manipulated. In these manipulated questions, the judges are provided false information about what they had initially answered, and what is presented as advice is in fact their own initial opinion. If the manipulation remains undetected, participants will thus have to decide how much they should accept what they think is a piece of advice but is in fact their own previous opinion.

## Experiment 1: Arbitrary answer vs. own initial answer

In Experiment 1, the manipulation was performed only on one question (question 6 out of 8). For this question, instead of being truthfully reminded of their own initial answer, the judges were provided with an arbitrary answer that was still described as their own. The advice presented as being from another participant was in fact their own initial answer. For this manipulated question, and for the participants who did not detect the manipulation, the predictions of the different explanations are as follows. Rational access to reasons predicts that judges should favor the advice (i.e. the judge’s initial answer), since they should access the reasons that had initially led them to provide this answer. Other explanations for conservatism, such as egocentric beliefs or biased access to reasons, predict that participants should favor the answer attributed to them that they think is their own prior opinion.

### Participants

We recruited 99 participants (39 females, *M*age = 33.7, *SD* = 10.1) residing in the United States of America through Amazon Mechanical Turk website. In all the present experiments, the sample sizes were chosen to be broadly in line with the sample sizes used in the advice taking literature (for reviews, see [6,7]). The experiments took about 6 minutes to complete, and we paid the participants standard rates for participation ($0.5). For all the experiments, data were analyzed anonymously, the institutional review board waived the need for written informed consent from the participants; only a short description of the experiment was provided to the participants at the very beginning. The studies were approved by the Lund University Ethics Board (Dnr: 2009/105). All the data is available (S1 File), and is strictly anonymous.

### Materials

Stimulus materials were questions about life expectancies of eight different countries (in a fixed order). We considered as a good answer the life expectancy estimated by the CIA in 2013. The countries we used, along with the good answer in brackets, were the following: Autralia (82), China (75), South Africa (49), Japan (84), India (67), Brazil (70), Russia (70) and USA (79). We used these numbers as advice.

### Procedure

The experiment consisted of two phases. In Phase 1, for each country we asked participants to place a slider from 0 to 100 according to their best estimate. At the start of Phase 2, which took place right after the Phase 1, we told the participants that “We will now proceed to the second phase of the experiment. For each of the eight countries, we will give you the answer given by another participant. Each answer was provided by a different participant. You will be able to change your answer in light of this information if you wish”.

**Fig 1:**
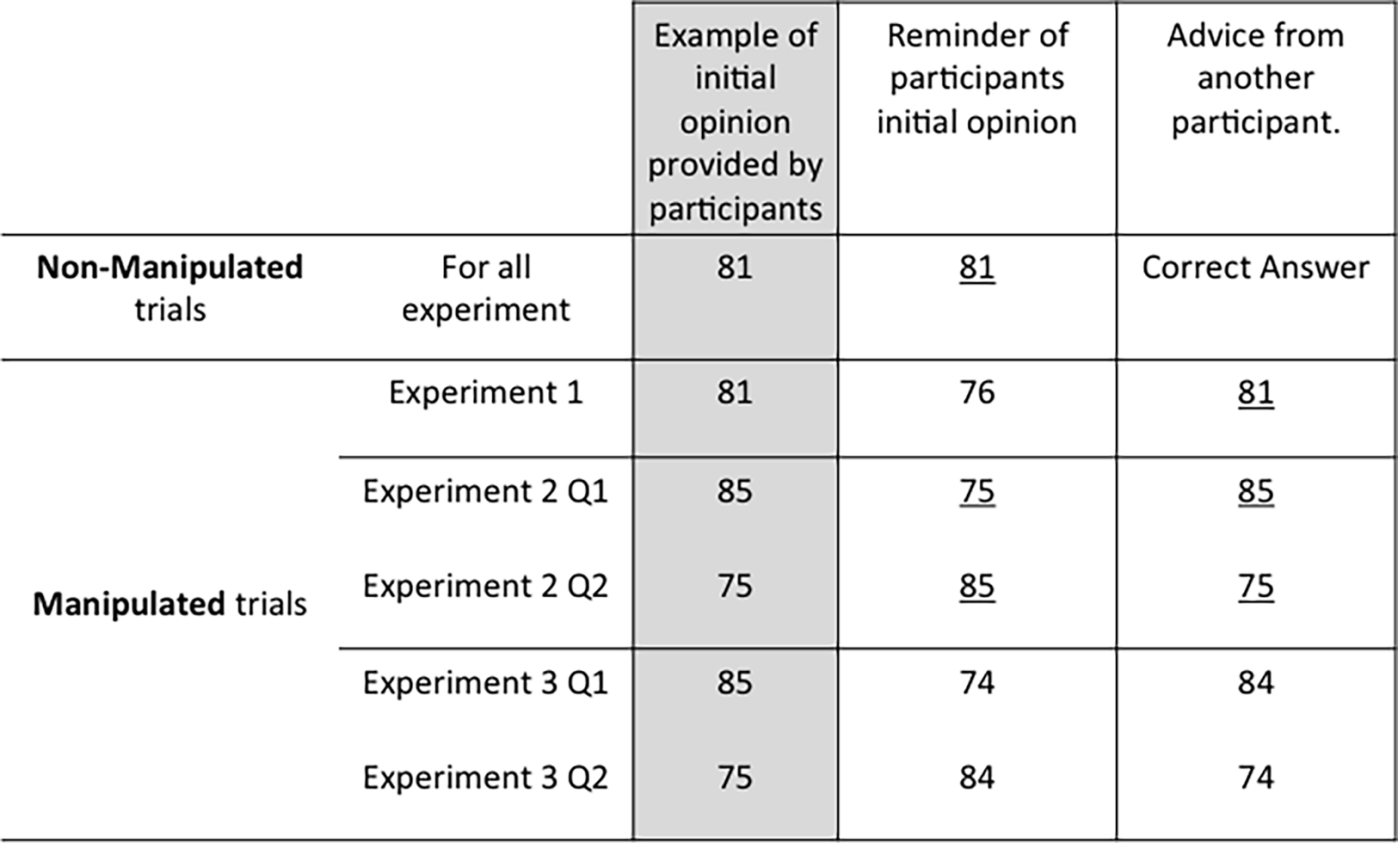
Design of the experiments. Experiment 4 had the same design as Experiment 2. Fluent choices are underlined. Non-Manipulated questions were the same for each experiment, only varying in number (7 in Experiment 1, 6 in Experiments 2 to 4).

For seven out of the eight questions, the participants were reminded of their own previous answer and provided with someone else’s answer. For the sixth question (the manipulated one), instead of being truthfully reminded of their previous answer, participants were only told that they were reminded of their previous answer. In fact, this answer was not the one they had given. Instead, depending on which of the following set the participant’s initial answer fall into: [0; 40], [41; 49], [50; 59], [60; 69], [70; 79], [80; 89], or [90; 100], we attributed as a previous answer respectively: 47, 57, 67, 76, 67, 76 or 87. The external features of the presentation were strictly identical to those of the other seven questions. See Fig 1 for a summary of the procedures for all experiments.

At the end of the experiment, we asked the participants a series of questions to determine if they had detected the manipulation of their answer. The first question simply asked what they thought of the experiment, the second if they thought anything was strange with the experiment, and in the third they were presented with an actual description of the manipulation and asked if they had noticed this: “During the second phase, in one or more question, your previous answers had been changed to another answer, and the "someone else's answer" were actually the one you had previously given.” If the participants answered yes, we asked them how many times they had noticed this, followed by a question asking them to specify which of the eight answers they thought had been altered. Finally, we asked the participants about their gender, age, and level of education.

### Results

#### Detection rates

When the manipulation procedure was described in the debriefing, 23% of the participants indicated that they had noticed their answers had been altered. All those participants were counted as detectors, even though 65% of them were unable to identify the country on which the manipulation took place. This suggests that the 23% measure is conservative, in that it probably includes participants who did not actually detect the manipulation. By contrast, it seems unlikely that the participants who said they had not detected the manipulation had in fact done so.

Using the self-reported 23% (Detectors) as a criterion of detection, we can be reasonably sure that the 76 participants classified as Non-Detectors were in fact unaware of the manipulation ([16,30] for discussions of participant awareness and detection criteria in choice blindness experiments,see [31]). Non-detectors treated their own estimation as if it was an advice from someone else. The following analyses bear on Non-Detectors (this will be true throughout the four experiments). This is also true for the results of the Non-Manipulated trials.

It is plausible that Detectors were, on average, more attentive than Non-Detectors, and that this difference might have influenced the results. However, given that three-quarters of the participants were Non-Detectors, our results cannot be said to bear on an overly specific population.

#### Data analysis

For each country and each participant, we calculated the weight participants placed in the advice. The participant’s final opinion (f) can be represented as a weighted combination of their own initial opinion (i) and the advice they received (a). The weight of advice (*WoA*) is defined as: | f–i | / | a–i | [see, e.g., 7]. In the case of Manipulated trials, *WoA* was calculated as follows: (f) was the final opinion of the participant (as in the Non Manipulated trials), (i) was the opinion attributed to the participants, and (a) was the opinion that was presented to them as advice. *WoA* is well defined if the final estimate falls between the initial estimate and the advice, as it did in 97% of the 792 total cases (including Detectors). The other cases were removed from the analysis.

Moreover, for the non-manipulated questions, when a participant gave the correct answer on their first estimate, that participant then faced a piece of advice that was the same as her initial opinion (since we used correct answers as advice). Sixteen such trials (2%) were also removed from the analysis.

*WoA* takes the value of 0 if the participant ignores the advice and doesn’t shift at all from his initial choice, and the value of 1 if the participant shifts completely towards the advice. In order to make a pairwise comparison of the *WoA* between non-manipulated and manipulated questions, on one side we calculated for each participant the mean *WoA* value over the seven non-manipulated questions and on the other side we took the *WoA* on the single manipulated question. We only report the between-subject standard deviations for each value.

#### Conservatism on Non-Manipulated questions

We excluded 29 (5%) trials because *WoA* was not well defined (for one of the two reasons detailed above). On average for the seven non-manipulated questions, *WoA* = 0.23 (*SD* = 0.20; *Mdn* = 0.18). One sample t-test indicated that this is significantly less than the *WoA* of averaging between the participants’ initial opinion and the advice (0.5) (*t*(75) = -11.4, *p* < .001, *d* = 1.3).

#### Conservatism on Manipulated questions

**Fig 2:**
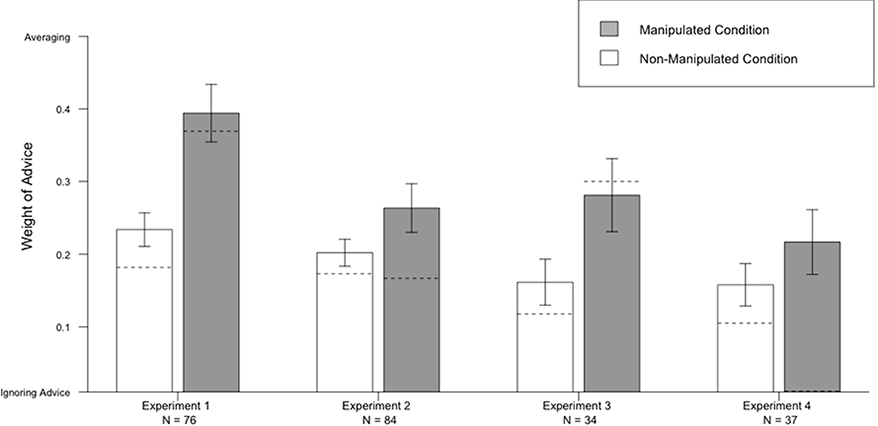
Results of Experiment 1 to 4. Weight of Advice in the Manipulated and Non-Manipulated questions of Experiments 1 to 4, only for participants who did not detect the manipulation. Standard error bars are displayed along with dotted lines representing the median answers.

We excluded 4 trials (5%) because *WoA* was not well defined. The average *WoA* for the manipulated question was 0.39 (*SD* = 0.3; *Mdn* = 0.37), significantly less than the *WoA* of averaging (*t*(71) = -2.7, *p* = .009, *d* = .3, 4 trials excluded). A paired t-test comparing non-manipulated and manipulated questions shows that participants have a greater *WoA* for manipulated than non-manipulated trials (*t*(71) = -4.5, *p* < .001, *d* = .58, 4 trials excluded). See Fig 2 for a summary of the results of all four experiments.

The results of the Non-Manipulated trials in Experiment 1 replicate the basic finding that participants are conservative: they put more weight on their own opinion than on a piece of advice. It expends on previous results by showing that participants are also conservative towards what they merely believe is their own opinion, in the Manipulated trials. However, conservatism was weaker on these trials than when participants were genuinely comparing their opinion to a piece of advice in Non-Manipulated trials.

These results are thus ambiguous. On the one hand, they suggest that rational access to reasons cannot explain conservatism entirely, since it predicts a complete reversal, with judges favoring the advice instead of the answer described as their own on manipulated trials. On the other hand, the results suggest that this explanation might be partially correct, since judges were less conservative on Manipulated problems than on Non Manipulated problems. On Manipulated trials, judges could have thought of reasons supporting the advice.

One factor that might explain the ambiguous result is repetition, a factor that has been largely ignored in the literature. In manipulated trials, the advice has already been processed by the participants—when they initially gave it as an answer. Repeated statements receive higher truth ratings than new statements, a phenomenon known as the *illusory truth effect* [32]. The illusory truth effect has been replicated many times, with different types of materials (for a recent example containing references to earlier work, see [33]). It is thus possible that, in Experiment 1, repetition could have created an illusory truth effect that would have drawn participants towards what was presented as a piece of advice but was in fact their own prior opinion.

## Experiment 2: Switch of initial answers and advice

To neutralize the effects of repetition, in Experiment 2 two questions were manipulated. In the second phase, the judge’s initial answer to question 4 was presented as the advice on question 4, while the answer to question 7 was presented as the judge’s initial answer to question 4. For question 7, the advice was in fact the initial answer to question 7, while the answer presented as the judge’s initial answer was in fact the answer to question 4.

The predictions are the same as for Experiment 1, except regarding the effects of repetition. For both Manipulated questions, repetition should have no impact, since both values—the answer described as the judge’s own and the advice—have previously been provided by the judges. However, on Non Manipulated questions the only repeated opinion is that previously provided by the participants, while the advice has not been repeated. Thus, repetition could not explain conservatism in the Manipulated trials, but it could explain a greater conservatism in Non Manipulated than in Manipulated trials.

### Participants

We recruited 100 participants (44 females, *M*age = 36.4, *SD* = 12.4) residing in the United States of America through Amazon Mechanical Turk website. The experiments took about 6 minutes to complete, and we paid the participants standard rates for participation ($0.5).

### Materials

The materials were identical to those of Experiment 1.

### Procedure

For half of the participants, the way they had to enter their estimations was identical to experiment 1: Participants were asked to place a slider both in the first and second phase of the experiment. For the other half of participants however, instead of using sliders, we asked them to enter their estimation manually (a format which will be used in a later experiment). None of the results reported here differed significantly between those two formats, hence we present them together.

### Results

#### Detection rates

When the manipulation procedure was described in the debriefing, 16% of the participants indicated they had noticed that their answers had been altered. Only 3 of them were able to identify the countries for which the manipulation took place. The following analyses only bear on 84 Non-Detectors.

#### Data analysis

Data analysis was essentially identical to that of Experiment 1, except that two questions are manipulated, hence the mean *WoA* for the non-manipulated is computed over six questions instead of seven, and the mean *WoA* for the manipulated is computed over these two questions.

#### Conservatism on Non Manipulated questions

Thirty eight trials (8%) were excluded because *WoA* was not well defined. The mean *WoA* for the non-manipulated questions was 0.20 (*SD* = .17, *Mdn* = .17), which significantly differ from the *WoA* of averaging (*t*(83) = -16.3, *p* < .001, *d* = .8). The current *WoA* was not significantly different from that of Experiment 1 (*t*(146.1) = 1.1, *p* = .28, *d* = .17).

#### Conservatism on Manipulated questions

Twenty five trials (15%) were excluded. The average *WoA* for the two manipulated questions was 0.26 (*SD* = .29; *Mdn* = 0.17), which significantly differ from the *WoA* of averaging (*t*(72) = -7.0, *p* < .001, *d* = 1.8, 11 trials excluded). The *WoA* was significantly larger for Manipulated than for Non-Manipulated trials (*t*(72) = -2.4, *p* = .02, *d* = .27). By contrast, the current *WoA* on the Manipulated questions was significantly smaller than the *WoA* on the Manipulated question of Experiment 1 (*t*(139) = 2.5, *p* = .01, *d* = .27).

#### Discussion

Experiment 2 supports the interpretation of the results of Experiment 1. Participants exhibited a strong conservatism (i.e. low *WoA*) on Non-Manipulated problems. This cannot be explained by rational access to reasons, since the reasons the participants had to form their initial opinion should now favor the advice, and thus yield a high *WoA*. These results are also compatible with a moderate effect of repetition, in two ways. First, on the Manipulated trials of Experiment 1, repetition favored the advice, while it favored equally the prior opinion and the advice in Experiment 2. This might account for the fact that, in the manipulated trials, participants put more weight on the advice in Experiment 1 than in Experiment 2. Second, in Experiment 2 repetition favored the prior opinion more strongly on Non-Manipulated trials (where it faced non-repeated advice) than on Manipulated trials (where it faced repeated advice). This might explain why people put more weight on their prior opinions in Non-Manipulated than on Manipulated trials.

## Experiment 3: Switch of initial answers and advice, repetition manipulation

Experiment 3 is a conceptual replication of Experiment 2 which uses another way to neutralize the effects of repetition on Manipulated trials. It has exactly the same design as Experiment 2 except that before being displayed in Phase 2, 1 was subtracted from the values of the answers (both the answer attributed to the participants and the answer presented as advice) to questions 4 and 7. As a result, neither answer was perceptually fluent in Phase 2.

### Participants

We recruited 50 participants (21 females, *M*age = 32.6, *SD* = 11.2) residing in the United States of America through Amazon Mechanical Turk website. The experiments took about 6 minutes to complete, and we paid the participants standard rates for participation ($0.5).

### Materials

Stimulus materials were exactly the same questions used on the first two experiments.

### Procedure

It has exactly the same design as Experiment 2 except for the modification noted above.

### Results

#### Detection rates

When the manipulation procedure was described in the debriefing, 32% of the participants indicated that they had noticed their answers had been altered. None of them were able to identify the countries on which the manipulation took place. The following analyses only bear on 33 Non-Detectors.

#### Data analysis

Identical to that of Experiment 1.

#### Conservatism on Non Manipulated questions

Six trials (3%) were excluded because *WoA* was not well defined. The average *WoA* for Non-Manipulated questions was 0.17 (*SD* = .19; *Mdn* = .13) which significantly differ from the averaging *WoA* (*t*(32) = -10.3, *p* < .001, *d* = .1.8).

#### Conservatism on Manipulated questions

Two trials (3%) were excluded because *WoA* was not well defined. The average *WoA* for the manipulated questions was 0.29 (*SD* = .29; *Mdn* = .34), which significantly differs from the averaging *WoA* (*t*(31) = -4.12, *p* < .001, *d* = .72, 1 trial excluded). The *WoA* was significantly larger for Manipulated than Non-Manipulated trials (*t*(31) = 2.9, *p* = .007, *d* = .51). By contrast, the current *WoA* on the Manipulated questions was smaller than the *WoA* on the Manipulated question of Experiment 1 but not significantly so (*t*(68.9) = 1.61, *p* = .11, *d* = .32).

#### Discussion

The results of Experiment 3 should be seen as a conceptual replication of those of Experiment 2, and supporting the same conclusion, namely, that rational access to reasons doesn’t seem to explain conservatism in the present experiments, and that the results so far are consistent with some effects of repetition.

## Experiment 4: Switch of initial answers and advice, speeded response

For the Manipulated questions of Experiments 1, 2, and 3, rational access to reasons cannot explain conservatism, but biased access to reasons can. One prediction of biased access to reasons is that conservatism should emerge only when judges have time to think of reasons, and that it should be stronger as judges have more time to access reasons (see, e.g. [34–36]). By contrast, egocentric beliefs and vigilant conservatism predict that conservatism should be the judges’ immediate reaction. To test this prediction, Experiment 4 replicates Experiment 3 while asking participants, in Phase 2, to answer as quickly as possible.

### Participants

We recruited 50 participants (13 females, *M*age = 35.0, *SD* = 11.3) residing in the United States of America through Amazon Mechanical Turk website. The experiments took about 6 minutes to complete, and we paid the participants standard rates for participation ($0.5).

### Materials

Stimulus materials were identical to those of Experiment 3.

### Procedure

The procedure was identical to that of Experiment 3 except that before starting the experiment, participants were asked to answer “as quickly as possible for every question”.

### Results

#### Detection rates

When the manipulation procedure was described in the debriefing, 26% of the participants indicated that they had noticed their answers had been altered. Only one of them was able to identify the countries on which the manipulation took place. The following analyses only bear on 37 Non-Detectors.

#### Reaction times

In order to offer an appropriate comparison of reaction times, the following analysis includes all participants of Experiment 4 and half of the participants of Experiment 2 (only those who had to enter their answers manually in phase 2). Outlier reaction times trial (more than three standard deviation away from the overall sample average, i.e larger than 16.7 seconds) were excluded. A log transformation was applied in order to make both distributions normal (Shapiro-Wilk normality test; Experiment 4, *W* = 0.97, *p* = .31; Experiment 2, *W* = 0.97, *p* = .25). Participants answered significantly faster in Experiment 4 (*M* = 5.4 sec, *SD* = 1.96) than in Experiment 2 (*M* = 6.34 sec, *SD* = 1.74; t-test on log-transformed data, *t*(75) = -2.58, *p* = .012, *d* = .57).

#### Conservatism on Non Manipulated questions

Twelve trials (5%) were excluded because *WoA* was not well defined. The average *WoA* for the Non-Manipulated questions was 0.16 (*SD* = .18, *Mdn* = .11) which significantly differs from the averaging *WoA* (*t*(36) = -11.7, *p* < .001, *d* = 1.92).

#### Conservatism on Manipulated questions:

Fifteen trials (20%) trials were excluded because *WoA* was not well defined. The average *WoA* for the Manipulated questions was 0.22 (SD = .25; Mdn = 0) which significantly differs from the averaging *WoA* (*t*(30) = -6.4, *p* < .001, *d* = 1.14, 6 trials excluded). The *WoA* was not different in Non-Manipulated and Manipulated trials (*t*(30) = 1.2, *p* = .23, *d* = .27). We observed a strong conservatism, not inferior to that of the Manipulated questions of in Experiment 2 (*t*(65.1) = -0.84, *p* = .40, *d* = .17).

#### Relation between reaction times and *WoA*

Even though the reaction times were shorter in Experiment 4 than in Experiment 2, they might still have been long enough for participants to find some reasons supporting the answers attributed to them. If the generation of these reasons explained conservatism, we should expect participants who take longer to answer to have a larger *WoA*. In fact there was no relation between reaction times and *WoA*. We used a Linear Mixed Model with (log-transfomed) response time as the dependant variable and random effect for subject and item, looking at the fixed effects of WoA and Condition (Manipulated or not). We found no main effect of WoA: *BetaWoA]* = -.13, *t*(217) = -.75, *p* = .46, nor interaction: *BetaWoAxCondition]* = .09, *t*(212) = .44, *p* = .66).

#### Discussion

Even when people provide rapid answers, they still have a low *WoA*. They do not discount advice less than when there is no time pressure. Moreover, *WoA* does not increase with reaction times. These results suggest that conservatism is not due only to biased access to reasons. Instead, it suggests that conservatism is the basic, immediate reaction when confronted with an opinion that conflicts with one’s own opinion (or what one thinks what is one’s opinion). This result is predicted both by egocentric beliefs and vigilant conservatism.

## Supplementary analyses

The present data can be mined to further test whether proximal explanations for conservatism could account for our results. In the following analyses, the data from Experiments 2 and 3 are aggregated to yield a larger data set. Experiments 1 and 4 were sufficiently different from 2 and 3 that we thought it would be more prudent not to aggregate all the data together.

### Performance

**Fig 3:**
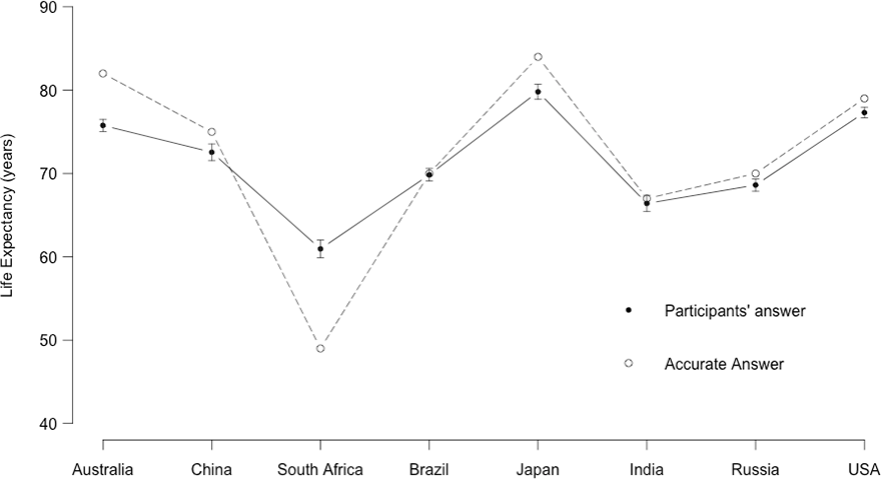
Country-by-country performance in Experiments 2 and 3. The white dots represent the correct answers, and the black dots the mean answers provided by the participants, along with standard error bars.

The relatively short reaction times, even in the absence of time pressure, could lead one to think that participants were essentially guessing. Conservatism could then be explained as the outcome of participants following a lazy strategy of typing back any available number. Besides the fact that this explanation wouldn’t account for why participants would tend to adopt their estimates rather than that of the advisor, the data shows that participants (Non-Detectors) performed reasonably well on the task (absolute error *M* = 8.1 years, *SD* = 7.0, *Mdn* = 6). The country-by-country pattern of answer also shows that participants were not answering in the same way for all countries (see Fig 3). This suggests that participants were, on average, taking the task seriously enough.

### Relation between performance and conservatism

The egocentric beliefs explanation for conservatism is that participants tend to think they are better than the average, and that this is why they tend to discount advice. To the extent that there is a correlation between actual performance and beliefs in one’s own performance (and there seems to be, see, e.g. [37,38]), this explanation entails that there should be a positive correlation between performance and conservatism. A linear regression analysis with the Non-Manipulated problems of Experiment 2 and 3 shows that there was no relation between the mean *WoA* and the mean absolute error of each subjects (*b* = -0.03, *t*(116) = -0.305, *p* = .76).

## General discussion

In four experiments, participants were first asked to provide an opinion on a series of questions (about life expectancy in different countries). In a second phase, participants were reminded of their initial opinion, provided with the opinion of an advisor, and asked to provide a new opinion. In each experiment, some of the opinions provided in the second phase were manipulated.

In Experiment 1, for one question participants were attributed an arbitrary opinion instead of their real prior opinion. On this question, what was presented as a piece of advice was actually the participant’s own prior opinion. Participants put more weight on the opinion arbitrarily attributed to them than on their own prior opinion. However, their bias towards the opinion they thought to be their own was weaker than their bias towards their real opinions (on Non-Manipulated questions). This difference could be an effect of repetition. On the Manipulated question, the advice was fluent while the attributed prior opinion was not. The reverse was true for Non-Manipulated questions.

Experiment 2 addressed the issue of repetition in the following way. Instead of attributing an arbitrary answer on one question, we relied on answers provided by the participant. The answers to one pair of questions were presented both as advice coming from someone else (as in Experiment 1), but also as the prior opinion of the participant on the other question in the pair. Thus, for these two questions, both the prior opinion (which was in fact the prior opinion on another question) and the advice (which was in fact the prior opinion on the same question) were equally fluent. On Manipulated questions, participants showed a strong conservatism in favor of the opinion they thought was theirs. Experiment 3 replicated the findings of Experiment 2 with another way of neutralizing the effects of repetition on Manipulated questions.

Experiment 4 tested the predictions of two explanations for conservatism: vigilant conservatism and biased access to reasons. Experiment 4 replicated Experiment 2 with one modification: in the second phase, participants were asked to answer as quickly as possible. As a result, participants would have either less time to generate reasons than in Experiment 2, or not time at all to do so. Biased access to reasons should thus predict a reduced or absent conservatism. Results revealed that, on the Manipulated problems, participants were still conservative—indeed, they were even non-significantly more so than in Experiment 2. Moreover, participants who took more time to answer were not more conservative.

Supplementary analyses offered further arguments against standard proximal explanations for conservatism. First, these analyses revealed that in spite of short reaction times, participants did not venture random guesses, showing that they took the task seriously, and thus that conservatism is unlikely to be the result of sheer laziness. Second, we found no relation between performance and conservatism. To the extent that actual performance and beliefs about one’s ability are correlated, this argues against the egocentric beliefs explanation.

Combined with the previous results reviewed in the introduction, these experiments suggest that people are biased towards their own opinion even when this bias cannot be accounted for by any of the proximal explanations developed so far in the literature (see [11]). In particular, the present experiments demonstrate that access to reasons—whether biased or rational—cannot be the only explanation for conservativeness in advice taking. This does not mean that these factors cannot make people more conservative, only that they would be adding to a more basic form of conservatism.

Even though vigilant conservatism is an ultimate explanation, it must be instantiated by proximal mechanisms. We suggested above that one crucial element of this proximal mechanism consists in checking the consistency between communicated information and one’s prior beliefs. Vigilant conservatism could then take place immediately following the detection of this inconsistency. This immediate reaction could then be overridden by other factors, such as trust in the source of the communicated information or arguments provided. The evidence briefly reviewed in the introduction suggests that such overrides are indeed effective. Still, more data will be needed to test specific predictions of vigilant conservatism.

These experiments also draw attention to a factor that had been largely neglected in the advice taking literature: repetition. Repetition has been shown to affect judgments of truth, so that repeated statements tend to be rated as more likely to be true (e.g. [32]). One should thus expect repeated advice to be taken into account more relative to non-repeated advice. This is what we observed in the comparison of Manipulated problems (for which the advice was not fluent) and Non-Manipulated problems (for which the advice was fluent) in Experiments 2 and 3. Also compatible with a role of repetition, conservatism was lowest when participants were faced with a non-repeated prior opinion and a repeated piece of advice, in the Non-Manipulated trials in Experiment 1.

Finally, regarding the role of internal and external cues to ownership, our present experiments add to the previous results of Harvey and Harries [4] by demonstrating that it is possible for external cues to ownership (i.e. what we tell participants is their opinion) to trump internal cues to ownership. In all four experiments, participants were consistently biased towards an opinion they thought was their own but was in fact not, against an opinion they thought was someone else’s but was in fact their own. The dissociation of internal and external cues to ownership enabled by the choice blindness paradigm could be used to further probe the functioning of vigilant conservatism.

## Supporting information

S1 File Data of all experiments. (ZIP)
